# Gut Microbiota Represent a Major Thermogenic Biomass

**DOI:** 10.1093/function/zqab019

**Published:** 2021-04-15

**Authors:** Ruth A Riedl, Colin M L Burnett, Nicole A Pearson, John J Reho, Mohamad Mokadem, Robert A Edwards, Tammy L Kindel, John R Kirby, Justin L Grobe

**Affiliations:** 1 Department of Pediatrics, Baylor College of Medicine, Houston, TX, USA; 2 Department of Internal Medicine, Division of Cardiology, University of Iowa Hospitals & Clinics, Iowa City, IA, USA; 3 Department of Laboratory Medicine and Pathology/Proteomics, Mayo Clinic, Rochester, MN, USA; 4 Department of Physiology, Medical College of Wisconsin, Milwaukee, WI, USA; 5 Comprehensive Rodent Metabolic Phenotyping Core, Medical College of Wisconsin, Milwaukee, WI, USA; 6 Division of Gastroenterology and Hepatology, Department of Internal Medicine, University of Iowa Hospitals & Clinics, Iowa City, IA, USA; 7 College of Science and Engineering, Flinders University, Adelaide, South Australia, Australia; 8 Department of Surgery, Medical College of Wisconsin, Milwaukee, WI, USA; 9 Cardiovascular Center, Medical College of Wisconsin, Milwaukee, WI, USA; 10 Department of Microbiology and Immunology, Medical College of Wisconsin, Milwaukee, WI, USA; 11 Genomic Sciences and Precision Medicine Center, Medical College of Wisconsin, Milwaukee, WI, USA; 12 Center for Microbiome Research, Medical College of Wisconsin, Milwaukee, WI, USA; 13 Neuroscience Research Center, Medical College of Wisconsin, Milwaukee, WI, USA

**Keywords:** energy, metabolism, microbiome, microbiota, gut

## Abstract

Evidence supports various roles for microbial metabolites in the control of multiple aspects of host energy flux including feeding behaviors, digestive efficiency, and energy expenditure, but few studies have quantified the energy utilization of the biomass of the gut microbiota itself. Because gut microbiota exist in an anoxic environment, energy flux is expected to be anaerobic; unfortunately, commonly utilized O_2_/CO_2_ respirometry-based approaches are unable to detect anaerobic energy flux. To quantify the contribution of the gut microbial biomass to whole-animal energy flux, we examined the effect of surgical reduction of gut biomass in C57BL/6J mice via cecectomy and assessed energy expenditure using methods sensitive to anaerobic flux, including bomb and direct calorimetry. First, we determined that cecectomy caused an acceleration of weight gain over several months due to a reduction in combined total host plus microbial energy expenditure, as reflected by an increase in energy efficiency (ie, weight gained per calorie absorbed). Second, we determined that under general anesthesia, cecectomy caused immediate changes in heat dissipation that were significantly modified by short-term pretreatment with dietary or pharmaceutical interventions known to modify the microbiome, and confirmed that these effects were undetectable by respirometry. We conclude that while the cecum only contributes approximately 1% of body mass in the mouse, this organ contributes roughly 8% of total resting energy expenditure, that this contribution is predominantly anaerobic, and that the composition and abundance of the cecal microbial contents can significantly alter its contribution to energy flux.

## Graphical abstract

**Figure:**
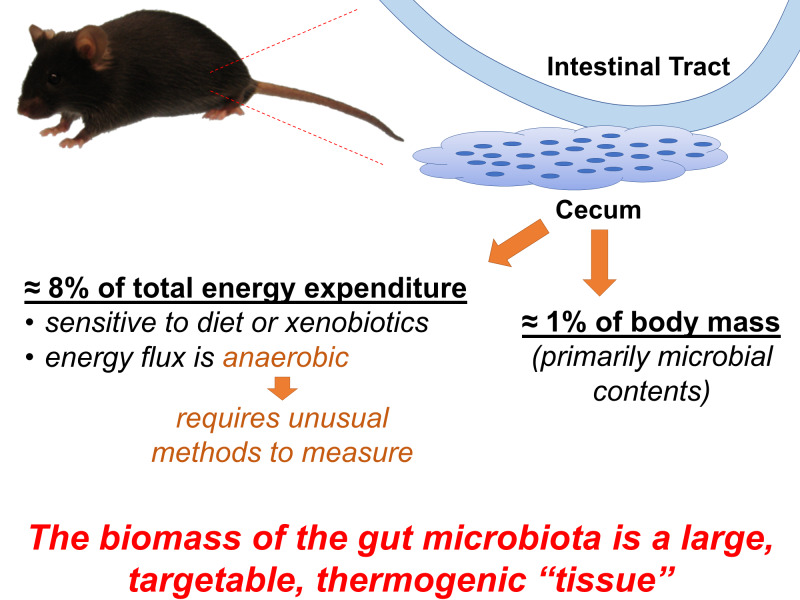

## Introduction

A role for resident bacteria within the gastrointestinal tract in metabolic physiology is well supported, and many studies have demonstrated that through production of an array of small molecule metabolites, bacteria “instruct” the physiology of the host.^1–4^ Often overlooked, however, is the fact that the gut microbiota represents a dynamic, energy-consuming biomass. It remains critically unclear how the mass and composition of this biomass contributes to total (ie, host + microbiota) energy flux, and therefore energy balance and weight gain or loss.

The biomass of the gut microbiota should, logically, contribute a physiologically and pathophysiologically significant fraction of total daily energy turnover. The NIH Human Microbiome Project estimated that microbiota constitute at least 1% of an adult human’s body mass, which for a 90 kg male, equates to 0.9 kg.^5^ Bacteria are estimated to generally exhibit a metabolic rate of approximately 7 kcal/kg/h,^6^ and therefore 0.9 kg of bacteria should consume on the order of 6.3 kcal/h, or 150 kcal/day (equal to 7.5% of a typical 2000 kcal/day total caloric flux).

The composition of the microbiota community should dictate the integrated energy flux of this biomass. Bacteria that can be grown in the laboratory in rich media display doubling times that reflect the conversion of calories from standard sources (eg, glucose, proteins) into biomass. While doubling times differ and are characteristic of any given bacterial species, growth rates must be determined empirically. For example, standard lab strains of *E. coli* typically used for cloning double every 20–30 min while other organisms that might be isolated from the environment (eg, *Myxococcus xanthus*) often display a doubling time of a few hours.^7^ The doubling time of a given organism is proportional to the turnover of nutrients, production of biomass, and generation of energy for the cells in the form of ATP. Because of the wide variety of growth rates found in vitro, and the differential association of unique gut microbiome community compositions with lean and obese states,^8^ we expect that organisms living in more complex environments such as the gut might display a wide range of consumption of calories.

Thus, we proposed the hypothesis that the gut microbiota contributes a disproportionately large fraction of resting energy expenditure relative to its biomass. Further, we hypothesized that the composition of this biomass (as dictated by diet or pharmaceuticals) significantly alters the contribution of this biomass to total resting energy expenditure. Importantly, to interrogate the role of gut microbiota in energy flux, unusual approaches were necessary because commonly employed O_2_/CO_2_ gas respirometry-based methods are unable to detect anaerobic metabolism in vivo.^9^ To assess total (ie, aerobic plus anaerobic) energy flux, bomb calorimetry and direct gradient–layer calorimetry methods were used to quantify the effects of surgical removal of the biomass of the gut microbiota (via cecectomy), within the context of dietary and pharmacological manipulations of the composition of this biomass. We determined that the gut microbiota biomass contributed a disproportionately large fraction of total energy flux, and that the composition of this biomass modifies its contribution to energy flux. As the mass and composition of the gut microbiota differ across development and in pathological states, these findings support the concept that the biomass of the gut microbiota represents a thermogenic “organ” and may represent a therapeutic target itself, to address energetic disorders from growth failures of prematurity through obesity in adulthood.

## Materials and Methods

Male C57BL/6J mice were obtained from Jackson Laboratories, maintained at 22°C on a 12:12 light cycle, and supplied Envigo 7913 chow unless otherwise specified. Body composition was serially determined across studies using time-domain nuclear magnetic resonance. All studies were approved by the University of Iowa and Medical College of Wisconsin Institutional Animal Care and Use Committees and conformed to the National Research Council’s Guide for the Care and Use of Laboratory Animals.^10^

One cohort of animals underwent a long-term study of the effect of cecectomy upon energy balance, using the surgical approach described by Jongwattanapisan.^11^ Before surgery, ingestive behaviors and quantitative fecal collections were performed using metabolic cages, as previously described.^12^ At 13 weeks of age, mice underwent aseptic surgical removal of the cecum or sham surgery under ketamine/xylazine anesthesia. Mice were then singly housed for the remainder of the study and underwent serial measures of food intake and fecal collection for the following 3 months. Food and fecal samples were then assayed for caloric content using a semimicro bomb calorimeter (Parr), to calculate digestive efficiency (the fraction of calories consumed that are absorbed by the animal and associated microbiota) and energy efficiency (the ratio of body plus microbial mass gains to calories absorbed).^12^

A second cohort of animals, 10–12 weeks of age, was treated for 1 week with the xenobiotic antipsychotic drug, risperidone (20 μg/mL in drinking water, which yields 80 μg/day^13^) before undergoing assessments of heat dissipation by combined calorimetry (gradient–layer direct calorimetry plus respirometry, described in detail previously^12^^,^^14^^,^^15^), before and after cecectomy. Mice were anesthetized by ketamine/xylazine, a radiotelemetric core temperature probe (DSI) was implanted inside the abdomen, and heat dissipation was determined using gradient–layer direct calorimetry within minutes of anesthesia induction.^9^^,^^12^^,^^14–16^ Upon establishment of a stable baseline, mice were removed from the calorimeter and underwent surgical removal of the cecum. Still under anesthesia, mice were immediately placed back into the calorimeter and metabolic rate was again determined. In a subset of animals, aerobic heat production was also estimated by O_2_/CO_2_ respirometry using the modified Weir equation.^12^^,^^17^

A third cohort of animals was switched to experimental diets at 9 weeks of age, for 1 week. While a set of animals were maintained on Envigo 7913 chow diet, other sets of mice were switched to a soy-free chow (Envigo 2920), a high fat/low carbohydrate diet (HFD; Research Diets D12451), or a high carbohydrate/low fat diet (HCD; Research Diets D12450H). At 10 weeks of age, animals underwent direct calorimetry under ketamine/xylazine anesthesia, as above, to assess heat dissipation before and after cecectomy.

Data were analyzed using parametric approaches throughout, including independent *t*-tests, ANOVA, and generalized linear modeling, followed by multiple comparison procedures, as indicated. For all analyses, two-tailed testing was performed and *P* < .05 was considered statistically significant.

## Results

### Surgical Mass Reduction of the Gut Accelerates Weight Gain via Suppression of Energy Expenditure

**Figure 1.**
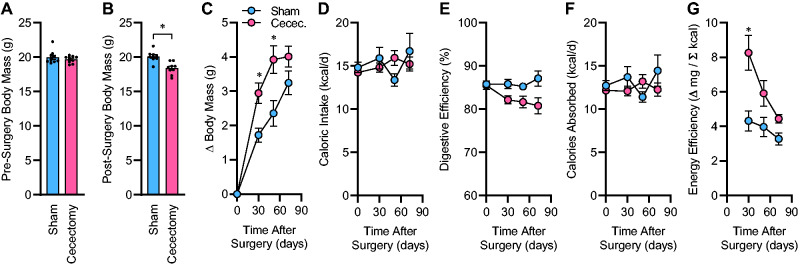
Cecectomy Causes Prolonged Increases in Energy Retention. (**A**) Body mass before surgery. (**B**) Body mass immediately after surgery. (**C**) Change in body mass after surgery. *P*_Surgery_=.008, *P*_Time_<.001, *P*_Interaction_=.007, *P*_Subject_<.001. (**D**) Calories ingested per day. *P*_Surgery_=.846, *P*_Time_=.421, *P*_Interaction_=.191, *P*_Subject_=.408. (**E**) Digestive efficiency. *P*_Surgery_=.014, *P*_Time_=.131, *P*_Interaction_=.030, *P*_Subject_=.001. (**F**) Calories absorbed per day. *P*_Surgery_=.403, *P*_Time_=.575, *P*_Interaction_=.166, *P*_Subject_=.250. (**G**) Energy efficiency. *P*_Surgery_=.005, *P*_Time_<.001, *P*_Interaction_=.014, *P*_Subject_=.001. For all panels, *n* = 9 male C57BL/6J mice per group, maintained on Envigo 7913 chow, with surgery performed at 13 weeks of age, and reported *P*-values calculated by two-way RM ANOVA. *P < .05 versus sham within time point, by independent *t*-test (B), or Bonferroni multiple comparisons procedure (C and G). Summary data are presented as mean ± SE.

Surgical removal of the cecum resulted in an expected immediate reduction in total body mass (Figure 1A and B). Following surgery, body mass gains of sham-treated mice were less than mice that had undergone cecectomy (Figure 1C). This accelerated weight gain in cecectomized mice was not due to changes in food intake behavior (Figure 1D). As expected, removal of the cecum resulted in a significant reduction in digestive efficiency (Figure 1E), but after correction of food intake for these changes in digestive efficiency, no change in the rate of caloric absorption was observed between groups (Figure 1F). As a result, the rate of weight gain per rate of caloric absorption, or energy efficiency, was significantly increased in mice after cecectomy compared to the sham group (Figure 1G). Energy efficiency represents an integrated, inverse metric of total energy output (ie, activity-dependent plus -independent mechanisms of the host plus microbial biomass).^16^ Together, these results indicate that surgical removal of the biomass of the cecum has prolonged effects to increase weight gain and that this is mediated by a suppression of energy expenditure but not through increased caloric ingestion or absorption. Importantly, because the energy flux of the animals progressively adapted over the months following surgery, it was difficult or impossible to quantitate the immediate, specific effect of the cecectomy surgery upon energy expenditure using the bomb calorimetry approach. Further, using this approach, it is not possible to dissect the relative contributions of activity-dependent versus -independent, and aerobic and anaerobic processes in this change in energy expenditure. Thus, additional complementary experiments using other methodologies were necessary.

### Xenobiotic Manipulation of the Gut Microbiota Alters the Energy Expenditure of the Cecum

**Figure 2.**
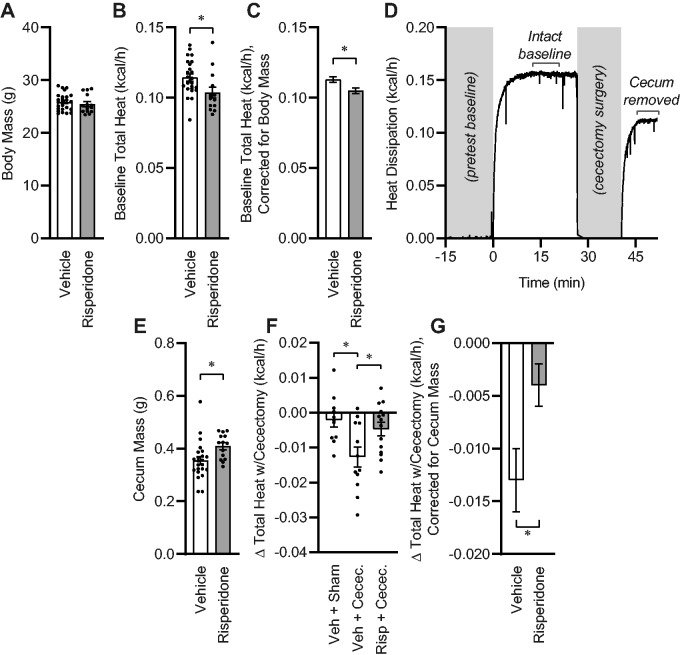
One-Week Xenobiotic Pretreatment Attenuates Energy Expenditure by the Cecum. (**A**) Body mass. (**B**) Total heat dissipation determined by direct calorimetry, under general anesthesia. (**C**) Estimated marginal mean heat dissipation at mean body mass of 25.72g. *P*_Model_<.001, *P*_BodyMass_<.001, *P*_Risperidone_=.010, *R*^2^=0.596. (**D**) Example tracing of total heat dissipation, illustrating time course of cecectomy experiment. (**E**) Cecum mass. (**F**) Change in heat dissipation immediately following cecectomy. (**G**) Estimated marginal mean change in heat dissipation immediately following cecectomy at mean cecum mass of 0.3888g. *P*_Model_=.049, *P*_CecumMass_=.282, *P*_Risperidone_=.016, *R*^2^=0.231. For all panels, *n* = 12 vehicle and *n* = 14 risperidone-treated male C57BL/6J mice. *P < .05 by independent *t*-test (B–D, F) or Tukey multiple comparisons procedure (E). Summary data are presented as mean ± SE.

Next, to more rapidly and specifically quantify the role of the composition of the gut microbial biomass in resting energy expenditure, we examined the effects of 2-week risperidone pretreatment upon total (ie, aerobic plus anaerobic) metabolic rate through the use of gradient–layer direct calorimetry. We employed risperidone-based manipulation of the gut, as we previously demonstrated a major role for risperidone in controlling both the composition of the gut microbiome and whole-body anaerobic energy expenditure. Importantly, we previously used fecal material transplant approaches to demonstrate the role of risperidone-induced shifts in community composition in these effects.^13^ As expected, short-term (2-week) exposure to risperidone had no effect on total body mass (Figure 2A), body fat (vehicle, 9.44 ± 0.60 vs risperidone 8.59 ± 1.04%), or hydration^18^ (total body water/body mass: vehicle, 66.33 ± 0.43 vs risperidone 66.93 ± 0.76%), however, heat dissipation was significantly reduced by risperidone, similar to our previous observations (Figure 2B).^13^ Correction of metabolic rates for individual body mass differences by generalized linear modeling underscored this conclusion, indicating that risperidone reduced heat dissipation by approximately 0.008 kcal/h, which represents 7% of baseline heat production (Figure 2C). To quantitate the metabolic contribution of the cecum and its contents, we removed the animal from the calorimeter and rapidly performed cecectomy (or sham) surgery. The animal was then immediately (ie, within minutes of the baseline recording) returned to the calorimeter chamber (Figure 2D). Consistent with an effect of risperidone upon the gut microbiota, risperidone increased the mass of the cecum (Figure 2E). Subsets of vehicle-treated animals underwent either sham surgery or cecectomy, and all risperidone-treated animals underwent cecectomy. Sham surgery had no effect on total heat dissipation, whereas cecectomy significantly reduced total heat dissipation in vehicle-treated animals (Figure 2F). In contrast, cecectomy had a significantly smaller effect on heat dissipation in animals that were pretreated with risperidone. Finally, correction of heat dissipation changes with cecectomy for individual differences in cecum masses by generalized linear modeling confirmed that risperidone reduced the effect of cecectomy on total heat dissipation by approximately 0.009 kcal/h (Figure 2G). Together, these data demonstrate that risperidone and cecectomy each independently reduce heat dissipation by roughly the same magnitude, and that these effects are nonadditive.

In a small number of animals, the change in aerobic heat production with cecectomy was estimated using O_2_/CO_2_ respirometry and the modified Weir equation. In contrast to the major effect of cecectomy on total heat dissipation as measured by direct calorimetry (Figure 2F), respirometry-based measures of gas exchange failed to estimate any effect of cecectomy on aerobic heat production in vehicle-treated animals (*n* = 5, −0.0035 ± 0.0029 kcal/h, *P* = .30 vs “no change” by one-sample *t*-test). Although cecectomy had a statistically significant effect on aerobic heat production in mice after risperidone pretreatment (−0.0055 ± 0.0020 kcal/h, *P* = .02 vs “no change”), this difference was indistinguishable from the change detected by direct calorimetry (−0.0047 ± 0.0019 kcal/h, *P* = .78), and these changes were substantially (43% and 37%, both *P* < .05) less than the reductions observed in animals with vehicle pretreatment (−0.0127 ± 0.0028 kcal/h, Figure 2F). We conclude that respirometry-based estimates of aerobic heat production are unable to detect the contribution of the gut microbiota to total heat production/dissipation, and by extension, that the contributions of the gut microbiota to total energy flux are anaerobic in form.

### Dietary Manipulation of the Gut Microbiota Alters Energy Expenditure of the Cecum

**Figure 3.**
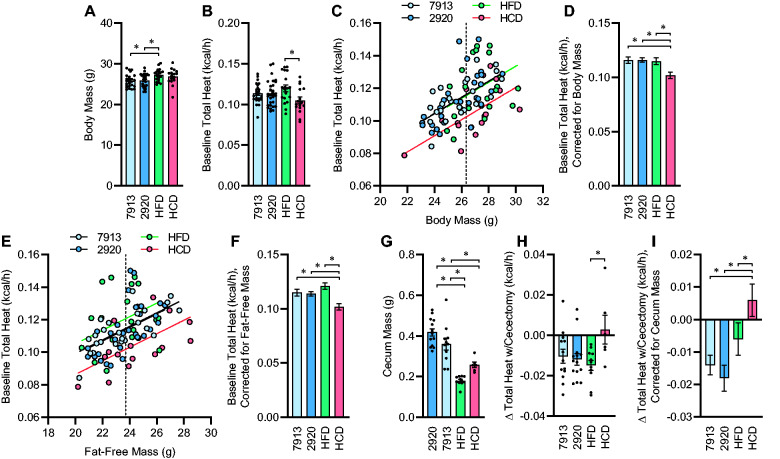
Energy Expenditure by the Cecum Is Dependent upon Diet. (**A**) Body mass. *P*_Diet_=.011. (**B**) Total heat dissipation determined by direct calorimetry, under general anesthesia. *P*_Diet_=.052. (**C**) Regression analysis of total heat dissipation versus total body mass. (**D**) Estimated marginal mean heat dissipation at mean body mass of 26.33 g, as indicated by dotted line in panel C. *P*_Model_<.001, *P*_BodyMass_<.001, *P*_Diet_=.005, *R*^2^=0.354. (**E**) Regression analysis of total heat dissipation versus fat-free mass. (**F**) Estimated marginal mean heat dissipation at mean fat-free mass of 23.71g, as indicated by dotted line in panel E. *P*_Model_<.001, *P*_FFM_<.001, *P*_Diet_=.001, *R*^2^=0.331. (**G**) Cecum mass. *P*_Diet_<.001. (**H**) Change in heat dissipation immediately following cecectomy. *P*_Diet_=.039. (**I**) Estimated marginal mean change in heat dissipation at mean cecum mass of 0.3198 g. *P*_Model_=.006, *P*_CecumMass_=.027, *P*_Diet_=.004, *R*^2^=0.313. For panels A–D, *n* = 24 fed Envigo 7913, *n* = 30 fed Envigo 2920, *n* = 19 fed Research Diets D12451 (HFD), and *n* = 16 fed Research Diets D12450H (HCD), and for panels E–G, *n* = 11 fed 7913, *n* = 15 fed 2920, *n* = 11 fed HFD, and *n* = 6 fed HCD. For all panels, reported *P*-values calculated by one-way ANOVA (A, B, G, and H) or univariate analysis of variance (D, F, and I). *P < .05 between indicated groups by Tukey multiple comparisons procedure. Summary data are presented as mean ± SE.

Finally, we examined the effects of 1-week dietary pretreatment upon the energy flux through the cecum. One week of HFD feeding caused a significant increase in body mass compared to both chow diets, while 1 week of HCD feeding did not have a statistically significant effect (Figure 3A). Total heat dissipation was significantly greater in HFD vs HCD mice, while chow-fed animals exhibited an intermediate phenotype (Figure 3B). Comparing total heat dissipation rates against body masses within groups illustrated a divergence in this relationship in mice fed HCD versus the other diets (Figure 3C). Correction for body mass using generalized linear modeling further illustrated this effect (Figure 3D). Similarly, comparing total heat dissipation rates against fat-free body masses also illustrated the suppressive effect of the HCD upon energy expenditure (Figure 3D–F). Cecal mass was significantly reduced in HFD and HCD groups compared to chow-fed groups (Figure 3G). Interestingly, while baseline heat dissipation was reduced in mice fed HCD (Figure 3C–F), cecectomy resulted in significant and similar reductions in heat dissipation in chow- and HFD-fed groups, but no consistent effect on heat dissipation in the HCD-fed group (Figure 3H). Correction of changes in heat dissipation rates for cecum mass was performed using generalized linear modeling, further illustrating the differential effect of cecectomy to reduce heat dissipation in chow- and HFD-fed but not HCD-fed mice (Figure 3I). Notably, whereas the mass of the cecum significantly influenced the impact of cecectomy upon heat dissipation, total body mass was not a significant covariate for the effect of cecectomy upon change in heat dissipation and its inclusion in the model did not qualitatively change any conclusions, so this covariate was removed from statistical analyses. Together, these data illustrate the differential effect of 1 week of diet switch upon the contribution of the biomass of the cecum (and its contents) to total body energy flux.

## Discussion

To execute these studies, two established (but infrequently used) methods were employed to assess energy flux: bomb calorimetry and direct calorimetry. These two methods were necessary because commonly-employed O_2_/CO_2_ respirometry methods are, by definition, unable to detect anaerobic metabolism.^9^ Fundamentally, Mansell and Macdonald demonstrated in 1990 that when other noncanonical fuels (eg, short-chain fatty acids and proteins) are utilized by an organism instead of typical fatty acids and simple sugars (eg, palmitate and glucose), the grossly oversimplified linear equations relating oxygen consumption to heat production become untenable.^19^ Thus, because gut bacteria live within an anoxic environment and are known to utilize a wide array of fuel sources, other methods such as direct calorimetry are required to quantitate their contribution to total (ie, host + microbiota) energy flux. Various studies from our and others’ laboratories since the mid-1970s using direct calorimetry in combination with gas respirometry have shown that lean humans, rodents, and various birds exhibit relatively large rates of energy flux that are anaerobic in form.^9^^,^^13–15^^,^^20–24^ Importantly, this flux has been demonstrated to be modified by sex, disease states (eg, obesity), diets, genetic manipulations, pharmaceuticals, gastric bypass surgeries, and manipulations of the gut microbiota (eg, fecal material transplants, bacteriophage transplant).^9^^,^^13–15^^,^^20–24^ Our group has also recently proposed the combinatorial use of body composition analysis and gas respirometry-based approaches to estimate anaerobic energy flux, and we documented major interactions between sex and diet upon this oft-ignored form of energy flux.^25^ Based on the results of the current study, we posit that the majority of the relatively large anaerobic energy flux that occurs in vivo is contributed by the gut microbiota. Unfortunately, very few laboratories utilize technologies that are capable of detecting and quantitating “non-aerobic” forms of energy flux, and no commercial sources of direct calorimeters currently exist. Development of turn-key systems of this type by forward-thinking engineering companies will greatly enable the future study of the physiological and pathophysiological significance of the gut microbiota and its anaerobic energy flux.

It is important to appreciate that debates regarding the accuracy of respirometry-based estimations in general,^17^^,^^19^^,^^26^ and respirometry- versus direct calorimetry-based methods of measuring in vivo energy flux,^9^^,^^16^^,^^27^^,^^28^ have been ongoing for many decades. While studies mentioned above document diet-, body composition-, sex-, genetic-, pharmaceutical-, and microbial-dependent differences in results when humans, rodents, and birds have been studied by both methods simultaneously,^9^^,^^13–15^^,^^20–25^ others have reported experiments that fail to detect differences in results between the two approaches. For example, notable studies from McLean and other field magnates have documented relatively tight concordance between gas respirometry and direct calorimetry results from studies of cattle (*Bos taurus*), a species appreciated for maintaining a large gut/rumen microbial biomass.^27^^,^^29^ What remains critically unclear, however, is how the composition of the rumen microbiome, the composition of the diet supplied, the body composition and sex of the cattle, and the common practice of administering somatic growth-promoting antibiotics may all contribute to the suppression of anaerobic heat production in such animals.^30^ Indeed, studies in mice and humans support the concepts that increased body fat,^14^^,^^22^^,^^23^^,^^25^ and antibiotic use^13^ are both associated with suppression of anaerobic heat production, and the current study supports the notion that diets with proportionately greater carbohydrate load may also serve to suppress anaerobic heat production. Thus, future work to understand the dynamic contribution of anaerobic metabolism to total body energy flux will require much more rigorous attention to factors that we now understand to have major effects on gut microbiota.

The magnitude of energy flux by the cecum documented here (ie, ≈7%–8% of total heat dissipation) is very large in two contexts. First, estimates of the magnitude of energy imbalance (ie, intake vs output) necessary to explain obesity in developed countries are on the order of 0.5%, or roughly 7 kcal/day.^31^ Thus, if the gut microbiota contribute 7% of total energy expenditure (which would equal 140 kcal/day for an adult human with a 2000 kcal/day turnover), then relatively minor reductions in anaerobic energy flux by this biomass could easily account, quantitatively, for human obesity. Alternatively, stimulation of anaerobic flux by this biomass, whether dietary, pharmaceutical, or surgical, could represent novel therapeutic approaches to obesity. Second, across all animals in the current studies combined (regardless of treatment group), cecal mass accounted for 1.3% ± 0.1% of body mass, yet contributed 7.9% ± 1.2% of total body energy flux. By extension, the gut microbiota consume proportionately large amounts of energy per mass per unit time, similar to other organs recognized for their high metabolism (eg, brain, kidney, and heart). Clearly, the physiological significance of anaerobic energy flux through the gut microbial biomass—independent from and in synergy with its instructive role to control various host tissues via metabolite production, release, and signaling—warrants more study.

Importantly, limitations of the current study and necessary directions of future study should be noted. First, access to combined calorimetry equipment represents a major challenge and therefore independent study replication by other investigators is needed but will likely take some time. Second, appreciation and quantification of the contribution of the cecum and its contents to total energy flux in vivo will require additional studies that utilize methods such as direct calorimetry but are preferably applied in the conscious state without an opened visceral cavity; sham surgery and control diet interventions cannot account for all variables involved. Third, many additional studies using advanced approaches such as germ-free animals and fecal material transplant approaches are required to better understand the various mechanisms that dictate the composition of the gut microbial community, and how compositional shifts contribute to energy flux through this biomass. Although many such experiments are needed to further this area, the current study provides a critical experimental quantification of energy flux through the biomass of the gut microbiota in vivo.

In summary, the role of the gut microbiota in the integrated control of energy flux is widely appreciated, but most studies have focused on the “instructive” role of the microbiota in modifying the biology of the host. In contrast, studies presented here support a disproportionately large (relative to its mass) contribution of the microbiota itself to energy metabolism. Collectively, these findings highlight that the biomass of the gut microbiota within the cecum contribute nearly 8% of total energy flux in the mouse, that this contribution is essentially all anaerobic in form, and that this contribution is highly sensitive to the composition of the cecal microbiota as dictated by diet or pharmaceuticals. We conclude that the gut microbiota represents a large, anaerobic, modifiable, thermogenic biomass.

## Conflict of Interest Statement

J.R.K. has submitted patents describing microbial targets of risperidone.

## Funding

This work was supported by the National Institutes of Health (HL134850, HL084207, HL140000, AI108255, HL007485), the American Heart Association (18EIA33890055), the VA Merit Review Program (I01 BX004774-01), the National Science Foundation (MCB-1244021), the University of Iowa Department of Internal Medicine and Fraternal Order of Eagles Diabetes Research Center (to M.M.), the University of Iowa Stinski Innovator Award (to J.R.K.), the American College of Surgeons George Clowes Career Development Award (to T.L.K.), and the Medical College of Wisconsin Research Affairs Committee Award (to T.L.K.), Cardiovascular Center Michael Keelan Jr. MD Research Foundation Award (to T.L.K.), and CTSI “Obesity” Ensemble (UL1TR001436).
